# Hyperbaric Oxygen Therapy for Late Radiation-Induced Duodenal Toxicity After Stereotactic Body Radiotherapy in a Patient with Cholangiocellular Carcinoma: A Unique Case Report

**DOI:** 10.3390/curroncol33080459

**Published:** 2026-07-30

**Authors:** Ivana Mikolašević, Petra Cotić, Sara Matulić Čubranić, Mario Franolić, Iva Skočilić, Tihana Salopek, Marin Golčić, Alojzije Košić, Laura Radošić, Blanka Josipović, Karla Lisica, Lea Juras, Sara Francetić, Ana Bešvir, Andrej Belančić

**Affiliations:** 1 Tumor Clinic, Clinical Hospital Centre Rijeka, 51000 Rijeka, Croatiasaramatulic@gmail.com (S.M.Č.);; 2 Department of Basic and Clinical Pharmacology, Faculty of Medicine, University of Rijeka, Braće Branchetta 20, 51000 Rijeka, Croatia

**Keywords:** stereotactic body radiotherapy, hyperbaric oxygen therapy, ulceration

## Abstract

Stereotactic body radiotherapy (SBRT) is an effective and increasingly utilized treatment modality for abdominal tumors, offering high rates of local control with generally acceptable toxicity profiles. Nevertheless, rare but severe late gastrointestinal complications, including radiation-induced ulceration, may occur and significantly impair patients’ quality of life, as well as continuation of oncologic treatment. Hyperbaric oxygen therapy (HBOT) has shown potential benefit in the management of chronic radiation-induced tissue injury, although evidence regarding its role following SBRT remains limited. We report the case of a 75-year-old woman with cholangiocellular carcinoma who developed severe radiation-induced duodenal ulceration following liver SBRT, presenting with persistent postprandial pain, nausea, vomiting, and substantial weight loss despite standard supportive treatment. Helicobacter pylori testing was negative, non-steroidal anti-inflammatory drug use was excluded, and histopathology showed chronic inflammatory and fibrotic mucosal injury with reactive epithelial changes. Despite high-dose proton pump inhibition, bismuth subcitrate, and nutritional support, symptoms and endoscopic ulceration persisted. HBOT was administered at 2.4 atmospheres absolute for 60 min over 30 sessions. Clinical improvement was noted after three sessions, and treatment was completed without adverse effects. Follow-up endoscopy demonstrated almost complete ulcer regression, with complete symptom resolution, improved oral intake, and a 10 kg weight gain. To the best of our knowledge, this represents the first reported case describing the successful use of HBOT as a potentially effective adjunctive treatment for severe radiation-induced duodenal ulceration following liver SBRT in a patient with cholangiocarcinoma. Although encouraging, this observation should be interpreted cautiously, and prospective clinical studies are needed to further evaluate the efficacy, safety, and optimal timing of HBOT in this setting.

## Simple Summary

Radiotherapy can provide good control over some abdominal tumors, but it may occasionally damage nearby digestive organs. We describe a 75-year-old woman with bile duct cancer who developed a large ulcer in the duodenum after highly focused liver radiotherapy. She had severe pain after eating, nausea, vomiting, loss of appetite, and marked weight loss, and standard ulcer treatment did not provide sufficient relief. She was therefore treated with hyperbaric oxygen, which involves breathing pure oxygen in a pressurized chamber. Her symptoms improved rapidly, she regained weight, and follow-up endoscopy showed almost complete healing of the ulcer. This case suggests that hyperbaric oxygen may be a useful supportive option when severe radiation-related injury of the upper digestive tract does not respond to conventional treatment.

## 1. Introduction

Stereotactic body radiotherapy (SBRT) is an advanced form of external beam radiotherapy that delivers highly precise, high-dose radiation to extracranial tumors in a small number of fractions [1]. Through careful target delineation, immobilization, and image guidance, SBRT achieves a high tumor dose while minimizing irradiation of surrounding healthy tissues [1]. Its antitumor effect results mainly from direct DNA damage, but vascular and immune-mediated mechanisms may also contribute [1,2]. Within the abdomen and pelvis, SBRT is indicated for selected patients presenting with primary tumors, recurrent disease, oligometastases, or oligoprogression, particularly when surgery is not feasible [3,4,5,6]. The toxicity associated with SBRT is generally limited and site-specific, and it can present either acutely or late [1,6]. While uncommon, SBRT can cause serious gastrointestinal complications, which can result in bleeding, obstruction, or perforation, depending on the applied dose, location of the tumor, and patient-related factors. SBRT toxicity can present either acutely or late, and this is primarily driven by microvascular damage, chronic ischemia, inflammation, and progressive fibrosis, which can lead to ulceration, necrosis, or organ dysfunction [7,8]. Over time, these ulcers may progress to perforations or fistulas and lead to significant abdominal pain, diarrhea, nausea, and weight loss, all of which can negatively impact the outcome of patients [9]. However, while SBRT represents an effective and generally well-tolerated treatment option for selected abdominal and pelvic malignancies, providing high rates of local control in appropriately selected patients, treatment of late radiation injuries of the gastrointestinal tract is still limited [1,2,3,4,5,6].

Hyperbaric oxygen therapy (HBOT) has been used in the management of late radiation-induced tissue injury, particularly in chronic gastrointestinal, genitourinary, and soft-tissue complications. Its therapeutic effect is attributed to increased tissue oxygenation, promotion of angiogenesis, stimulation of fibroblast activity, and modulation of chronic inflammation in hypoxic and fibrotic tissues [10,11,12]. Clinical studies and reviews suggest that HBOT may alleviate symptoms and promote healing in selected patients with delayed radiation injury, including chronic gastrointestinal radiation toxicity [11,12]. However, the available evidence relates predominantly to complications following conventional radiotherapy, whereas data regarding HBOT for toxicities specifically associated with stereotactic body radiotherapy (SBRT) remain limited [11,12].

We present the case of a patient with cholangiocarcinoma who developed severe refractory duodenal ulceration after liver SBRT and achieved marked clinical improvement and near-complete endoscopic healing following HBOT, after unsuccessful treatment with other treatment modalities. To the best of our knowledge, there are no well-documented reports describing the use of HBOT for late radiation-induced complications following liver SBRT in patients with cholangiocarcinoma.

This case highlights a potentially novel therapeutic approach in this clinical setting, particularly in light of the increasing use of SBRT in the treatment of oncologic patients.

## 2. Case Presentation

A 75-year-old female patient with a medical history significant for arterial hypertension, gastroesophageal reflux disease, hiatal hernia, paroxysmal atrial fibrillation (status post-pulmonary vein cryoablation), migraines, and prior ovariectomy initially presented in April 2024 after an abdominal ultrasound revealed a cystic liver lesion. Subsequent contrast-enhanced computed tomography (CT) demonstrated a 48 × 43 mm heterogeneous mass in segment V of the liver, inseparable from the gallbladder, suspicious for cholangiocarcinoma.

Further evaluation at an external institution using ultra-high-definition 3T PET/MR demonstrated increased metabolic activity in two closely adjacent lesions within segment V, as well as in lymph nodes located in the hepatoduodenal ligament, suspicious for metastatic involvement.

In May 2024, the patient underwent stereotactic body radiotherapy (SBRT) at an external institution targeting both the primary lesion and the involved lymph node. Treatment was delivered using a RapidArc technique with multiple arcs and flattening filter-free (FFF) photon beams. A dose of 3 × 10 Gy was delivered to the involved lymph node, while a single fraction of 32 Gy was administered to the primary lesion. During the first course of SBRT, which targeted the primary tumor and a periportal lymph node, the mean dose (Dmean) to the duodenum was 7.8 Gy, while the maximum dose (Dmax) was 34.6 Gy.

Three days after SBRT, the patient underwent surgical resection involving liver segments V and VI, cholecystectomy, and lymphadenectomy at another institution. The rationale for the selected SBRT regimen and the decision to perform surgical resection three days later could not be clarified, as both treatments were planned and performed at another institution, and detailed treatment planning records were not available to the authors. Histopathological analysis confirmed moderately differentiated cholangiocellular carcinoma (G2), staged as pT2N0, with lymphovascular and perineural invasion present and clear surgical margins (R0).

At the first evaluation at our institution in June 2024, adjuvant chemotherapy was recommended; however, the patient declined systemic treatment and opted for active surveillance. No evidence of disease was observed until December 2024, when follow-up PET/MR revealed multiple new satellite liver lesions and widespread osteolytic bone metastases. Systemic treatment with cisplatin, gemcitabine, and durvalumab was initiated in January 2025. Due to nausea and vomiting, chemotherapy doses were reduced by 25% from the second cycle onward. Zoledronic acid was introduced, and palliative radiotherapy (8 Gy in a single fraction) was administered to painful bone metastases.

After 4 cycles of chemoimmunotherapy, a CT scan performed in April 2025 demonstrated stable disease. In April 2025, a second course of SBRT was delivered to three liver metastases with a prescribed dose of 54 Gy in three fractions. As these lesions were located more peripherally (subcapsular), the radiation exposure to the duodenum was minimal, with a Dmean of 0.78 Gy and a Dmax of 6.24 Gy.

**Figure 1:**
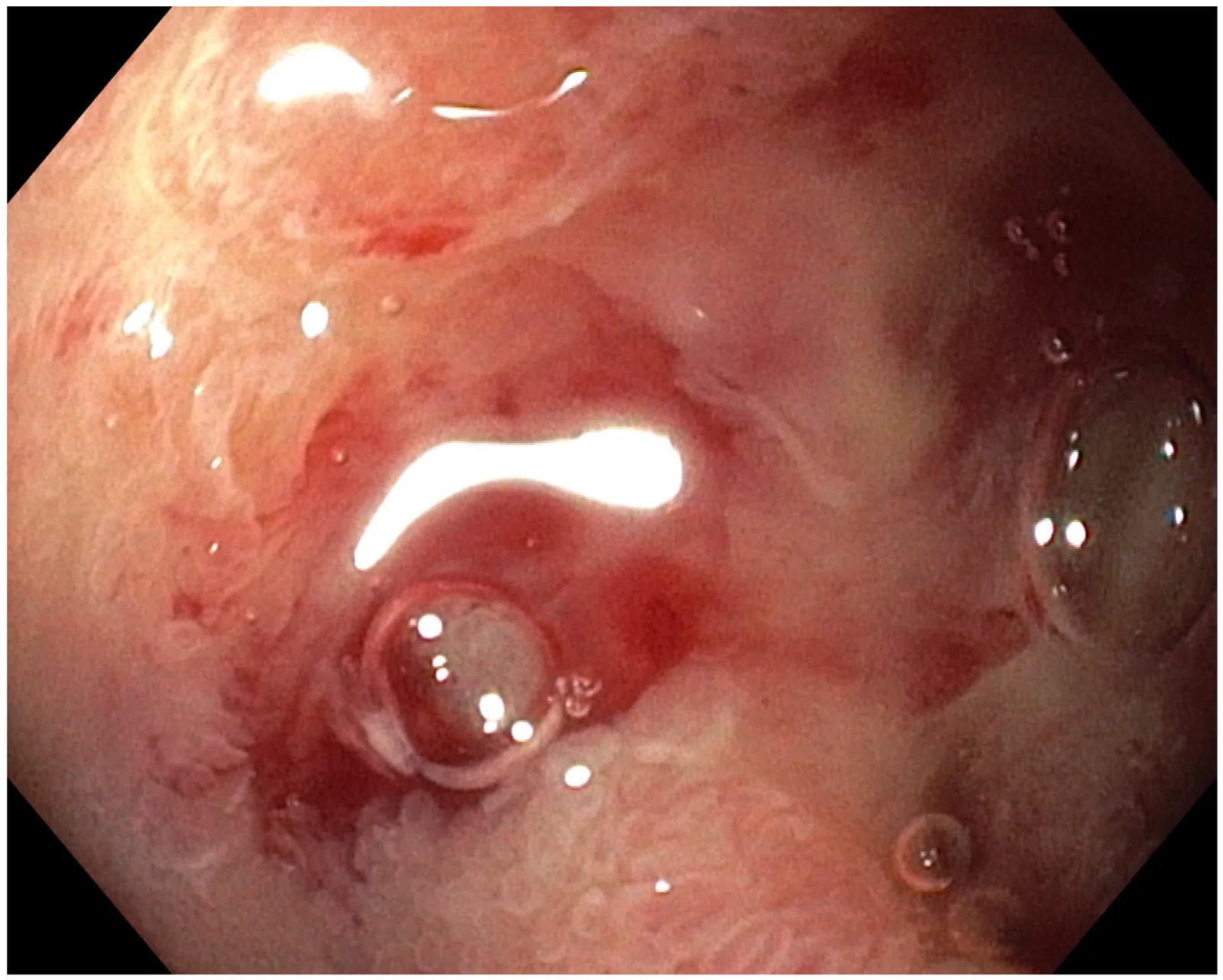
Endoscopic figure showing an extensive, deep ulcer with areas of focal bleeding at the transition from the duodenal bulb to the descending duodenum.

Approximately 6 months later, the patient was hospitalized at our institution due to epigastric pain, nausea, and generalized weakness. She denied the use of non-steroidal anti-inflammatory drugs, and testing for *Helicobacter pylori* was negative. Esophagogastroduodenoscopy (EGD) demonstrated extensive deep ulceration with areas of focal bleeding at the transition from the duodenal bulb to the descending duodenum (Figure 1). Biopsy samples were obtained, and histopathological analysis revealed inflammatory and fibrotic changes in the duodenal mucosa, with reactive epithelial alterations and varying degrees of atypia, consistent with chronic injury. The ulcer was classified as grade 3 according to the Common Terminology Criteria for Adverse Events version 5.0.

Given the history of prior SBRT, the findings were considered compatible with radiation-induced duodenal injury secondary to previous liver SBRT. Proton pump inhibitor therapy was initiated according to the institutional protocol. As there were no signs of perforation, surgical intervention was not indicated. Therefore, standard ulcer management was initiated, including high-dose proton pump inhibitor therapy and bismuth subcitrate. Because of poor oral intake related to persistent post-prandial pain and loss of appetite, the patient required both enteral and parenteral nutritional support to maintain adequate nutritional status and body weight. Follow-up CT imaging in October 2025 demonstrated regression of liver lesions with stable bone disease, and treatment was de-escalated to maintenance durvalumab with continued zoledronic acid. Despite supportive treatment, only partial clinical improvement was achieved. However, the patient continued to experience substantial symptoms, with progressive deterioration in performance status (ECOG 3), persistent weight loss, and markedly impaired quality of life despite comprehensive supportive therapy.

**Figure 2:**
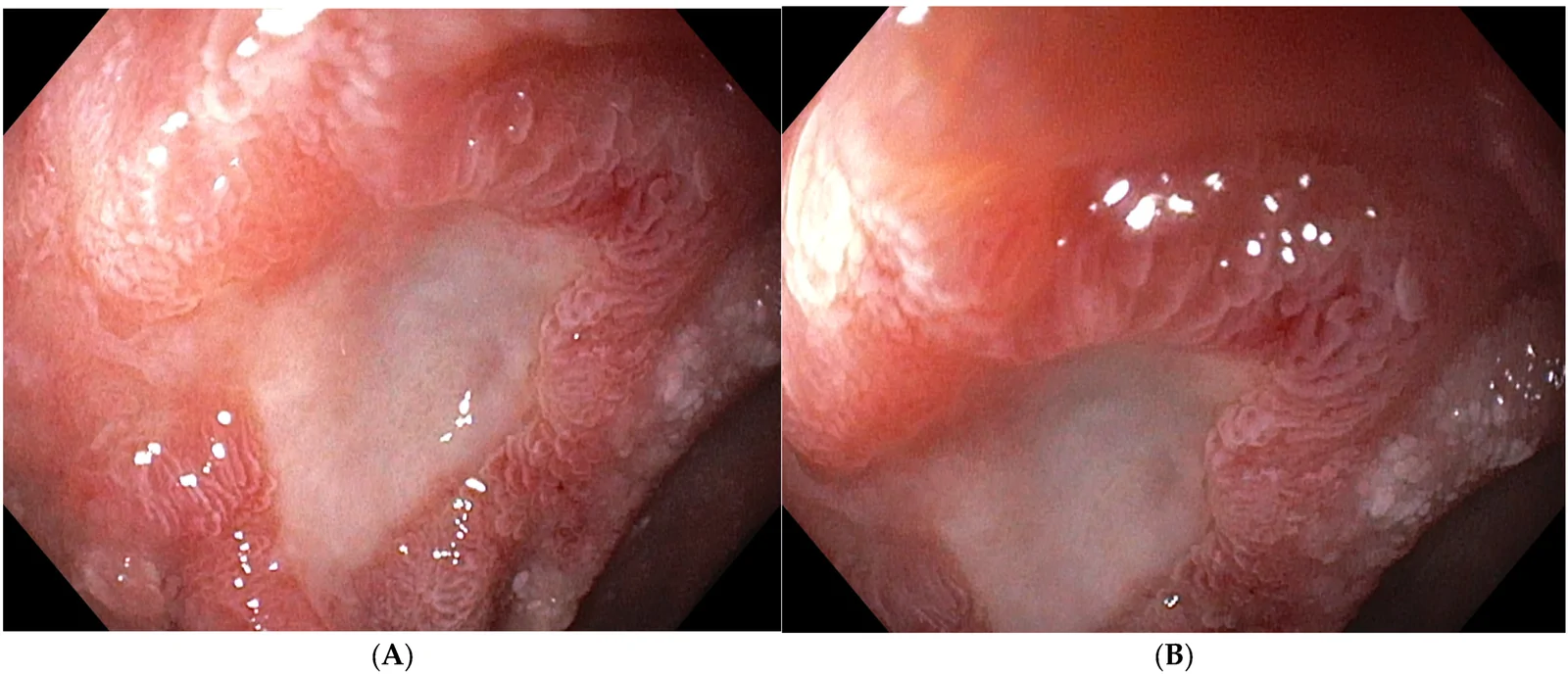
(**A**,**B**) Endoscopic images showing a persistent, extensive duodenal ulcer with a fibrin-covered base (Forrest III).

However, a repeat EGD in December 2025 demonstrated a persistent duodenal ulcer with a fibrin-covered base (Forrest III) (Figure 2A,B). Conservative management with pantoprazole (40 mg twice daily) and bismuth subcitrate therapy was continued.

Despite partial improvement, the patient experienced recurrent dyspeptic symptoms in January 2026, including epigastric pain after every meal, nausea, reduced appetite, weight loss, and general deterioration. As a result of these symptoms, she experienced a 15 kg weight loss. Given the presence of a chronic, refractory duodenal ulcer not responding to standard medical therapy, a multidisciplinary team, with the patient’s consent, recommended hyperbaric oxygen therapy as a salvage treatment for radiation-induced duodenal injury. After an appropriate pre-treatment evaluation, including spirometry, chest radiography, laboratory testing, electrocardiography, and pulmonology assessment, which revealed no contraindications, hyperbaric oxygen therapy was initiated on 28 January 2026. The treatment protocol consisted of 30 sessions, with each session carried out at 2.4 atmospheres absolute (ATA) for 60 min.

Clinical improvement was observed after the first three HBOT sessions, with a significant reduction in epigastric pain and improvement in appetite. The treatment was well tolerated, without any reported adverse effects.

**Figure 3:**
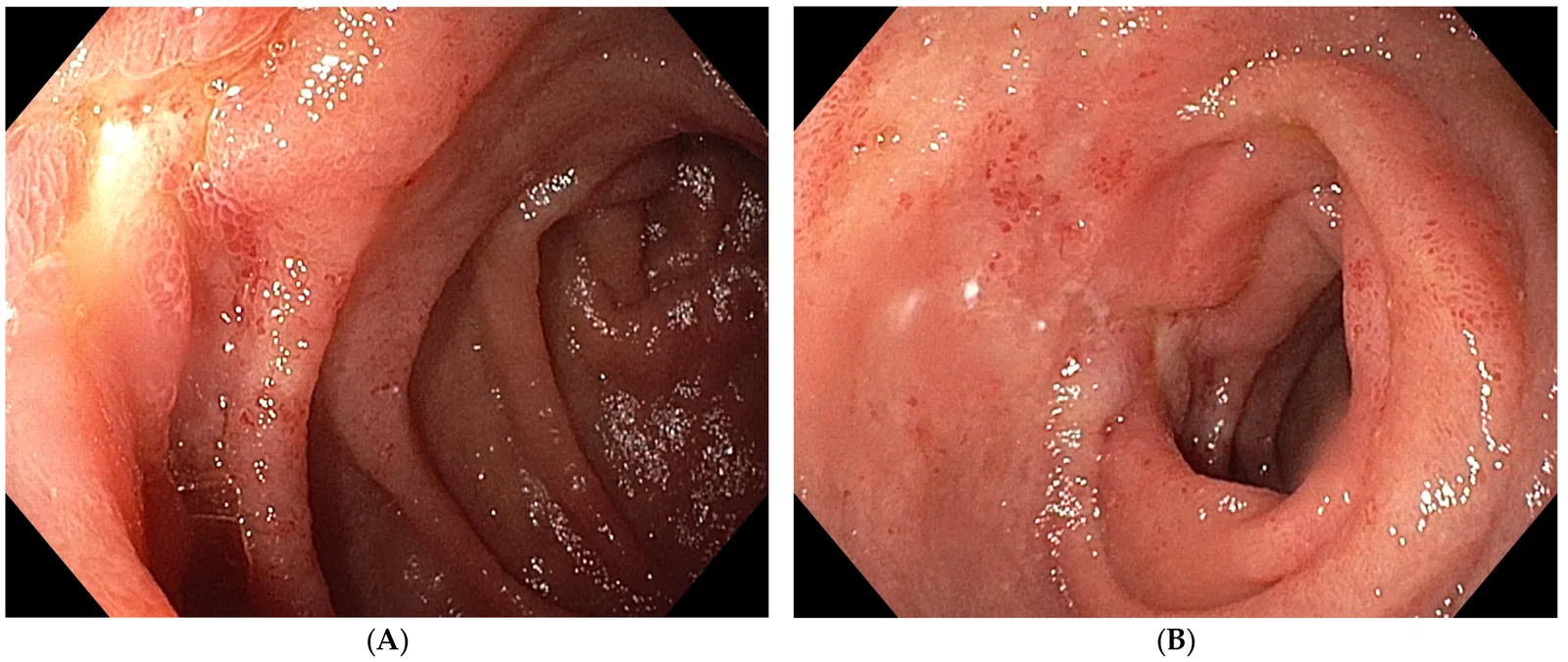
Esophagogastroduodenoscopy images after hyperbaric oxygen therapy treatment (**A**,**B**) demonstrating almost complete regression of the previously described large duodenal ulcer.

Follow-up esophagogastroduodenoscopy performed in March, 2026, demonstrated almost complete regression of the previously described duodenal ulceration (Figure 3A,B). By the completion of HBOT, the patient experienced complete resolution of symptoms, including postprandial epigastric pain, nausea, and vomiting, accompanied by normalization of oral intake and substantial weight recovery. Notably, the patient gained 10 kg following treatment.

These findings suggest a potential therapeutic role of HBOT in the management of refractory radiation-induced gastrointestinal toxicity, particularly in cases where conventional treatment approaches fail to achieve adequate clinical improvement.

**Figure 4:**
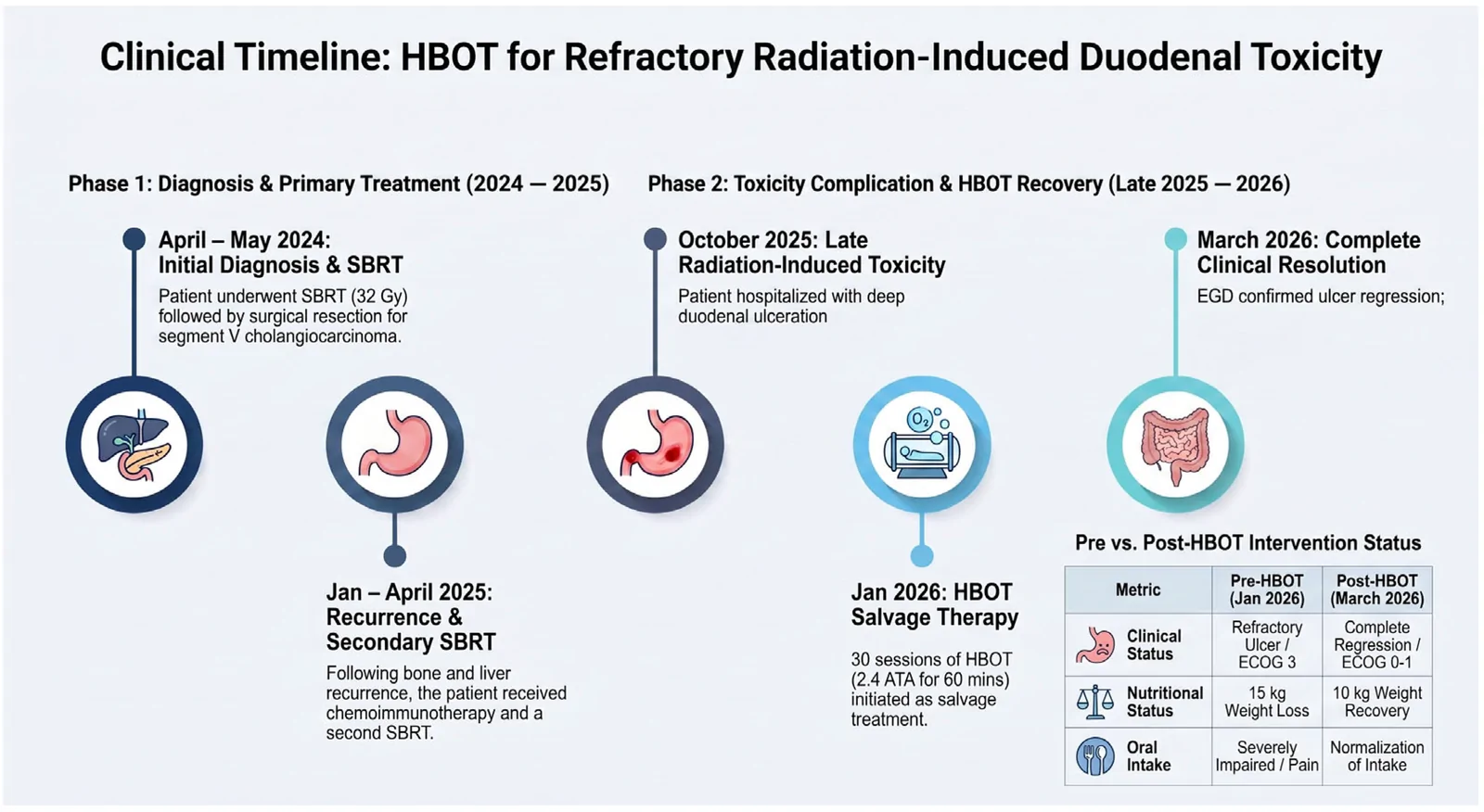
The case timeline.

Throughout hyperbaric oxygen therapy, maintenance systemic treatment with durvalumab and zoledronic acid was continued without interruption. Figure 4 demonstrates the case timeline.

## 3. Discussion

Radiotherapy is used in treatment in up to 50% of cancer patients and contributes to approximately 25% of cancer cures [10]. With growing interest in high-dose radiation therapy techniques, the toxicity burden of organs at risk (OARs) appears higher, which raises concerns regarding the adequate management and standardized care of SBRT complications. Gastrointestinal post-radiation toxicities are important factors to consider while planning for SBRT of abdominal and pelvic organs—the liver in our case [11,12,13,14,15]. Most acute radiation toxicities are mild and self-limited, usually resolving within 3 months [7]. On the other hand, late radiation toxicities are less common. For example, late radiation enteritis occurs in approximately 5% to 20% of patients after treatment. It may present with bleeding, ulceration, fistulas, strictures, and intestinal obstruction, usually 5 to 24 months following radiation [7]. In the case of our patient, post-radiation gastrointestinal toxicity manifested as an extensive and deep duodenal ulcer. Epigastric pain was the first symptom and was reported 6 months after the second course of SBRT for liver metastasis. Gastroduodenal ulceration has been reported as a potential late gastrointestinal complication following liver SBRT in patients with cholangiocarcinoma [11]. In our patient, SBRT was delivered on two separate occasions. As no esophagogastroduodenoscopy (EGD) findings obtained before October 2025 were available, it is not possible to determine with certainty whether the duodenal ulcer developed after the first or second course of SBRT. The maximum dose (Dmax) to the duodenum during the first SBRT course was 34.6 Gy. Published dose constraints for single-fraction liver SBRT generally recommend limiting the maximum dose to the duodenum to approximately 30 Gy or less, as higher doses are associated with an increased risk of severe gastrointestinal toxicity. Therefore, the recorded dose exceeded commonly accepted dose constraints. Together with the clinical presentation, the anatomical relationship between the target volume and the duodenum, and the histopathological findings consistent with chronic radiation injury, this supports radiation-induced duodenal ulceration as the most likely diagnosis. Although the exact timing of ulcer development cannot be established, the substantially higher duodenal dose delivered during the first SBRT course suggests that it was the more likely contributor to the patient’s late gastrointestinal toxicity.

The immune checkpoint inhibitor durvalumab, which our patient was receiving, is rarely associated with immune-mediated upper gastrointestinal inflammation, including gastritis and duodenitis [16,17]. However, in our patient, the endoscopic and histopathological findings were not considered typical of immune-mediated duodenitis. Endoscopically, ICI-associated duodenitis may present with diffuse or multifocal mucosal erythema and edema, loss of normal vascular patterns, granularity and friability of the mucosa, and erosions. Ulceration has also been described in more severe cases, but the finding is more often in the form of inflammatory duodenopathy/enteropathy rather than a single, isolated, deep, localized ulcer. Fold changes and an endoscopic appearance that may resemble celiac disease have also been described. In our patient, the lesion was highly localized, presenting as a large, deep postbulbar duodenal ulcer. Histologically, in the case of ICI-associated duodenopathy, active inflammation with neutrophils, intraepithelial lymphocytosis, increased numbers of lymphocytes and plasma cells in the lamina propria, cryptitis, and crypt abscesses are most commonly described. Epithelial apoptosis may be present, which is a particularly important finding in ICI-associated gastrointestinal injury. In some patients, villous blunting/atrophy is seen with increased numbers of intraepithelial lymphocytes, creating a celiac-like pattern. However, the histological pattern is heterogeneous and nonspecific and must be interpreted in the clinical context [16].

In our patient, the highly localized, deep postbulbar ulcer and the predominance of chronic inflammatory and fibrotic changes with reactive epithelial alterations were not considered typical of ICI-related duodenitis. Moreover, the duodenal ulcer in our patient developed in an anatomical region adjacent to the previously irradiated liver target. Additionally, the patient’s symptoms and endoscopic findings markedly improved during HBOT despite the continuation of immunotherapy and the lack of administration of immunosuppressive medications, which would represent the main treatment option for immunotherapy-related adverse effects. Previous data show that, following SBRT, grade ≥ 2 gastrointestinal adverse events, including gastritis and duodenitis, occur in 14–20% of patients, with these toxicities requiring significant consideration in SBRT planning for GI malignancies. For upper gastrointestinal tract injuries, both stenosis and ulcerations represent potential outcomes that can significantly affect patient outcomes [7,18,19,20]. On the other hand, a persistent, deep, highly localized postbulbar duodenal ulcer with chronic fibrotic histological changes is less characteristic of conventional chemotherapy-related gastrointestinal toxicity [21,22,23]. Hence, while we cannot absolutely exclude ICI duodenopathy or other etiologies of the ulcer, the endoscopic and histopathological patterns, together with the clinical course of our patient, favored chronic radiation-induced duodenal injury as the primary etiology.

HBOT increases tissue oxygenation by increasing the amount of dissolved oxygen in plasma, thereby improving oxygen delivery to hypoxic tissues. Re-oxygenation stimulates angiogenesis, improves fibroblast function and edema, and modulates inflammation. All these factors act against central pathophysiological mechanisms of chronic radiation injury and lead to tissue regeneration [12]. Earlier data suggested that, through the stimulation of angiogenesis, HBOT may promote tissue healing and represent an effective therapeutic option for almost two-thirds of patients with chronic gastrointestinal radiation injury. However, Marshall GT et al. [11] and Kernstine et al. [23] describe the beneficial effects of HBOT in gastrointestinal radiation toxicity following conventional radiotherapy rather than SBRT. The latest Cochrane review also notes that HBOT may improve outcomes in selected patients with late radiation tissue injury primarily involving the head and neck, bladder, and rectum [22]. However, no recommendation for gastric/duodenal tissue injury is given, which is why we believe that our case adds novel clinical evidence regarding the potential role of HBOT in the management of severe, refractory gastrointestinal toxicity specifically associated with SBRT. There is also evidence of promising therapeutic outcomes of HBOT in refractory peptic ulcer disease and proctitis, as well as bone and skin radionecrosis [13,24,25,26]. However, the optimal patient selection criteria, dose, and protocol remain to be elucidated.

In our case, the final EGD performed at the beginning of March 2026 demonstrated almost complete resolution of the duodenal ulcer, as well as the clinical symptoms of the patient. Furthermore, no contraindications were identified for administering planned oncologic treatment (durvalumab and zoledronic acid) during HBOT, so systemic antitumor therapy was continued in parallel. After 30 HBOT sessions, a significant clinical improvement was observed. The patient’s performance status improved to ECOG 1, her appetite increased, her abdominal pain diminished, and she gained approximately 10 kg of body weight.

To the best of our knowledge, this represents the first reported case describing the successful use of HBOT as a potentially effective adjunctive treatment for severe radiation-induced duodenal ulceration following liver SBRT in a patient with cholangiocarcinoma. While spontaneous or delayed ulcer healing under prolonged medical and nutritional management cannot be theoretically excluded, clinical improvement was rapid and noted immediately following HBOT. Hence, although encouraging, this observation should be interpreted cautiously, and prospective clinical studies are needed to further evaluate the efficacy, safety, and optimal timing of HBOT in this setting.

Importantly, certain limitations and contraindications regarding the concomitant use of HBOT and anticancer therapies have been described in the literature. In particular, specific chemotherapeutic agents, such as bleomycin and doxorubicin, may exhibit synergistic toxicity when combined with HBOT and therefore require careful consideration before treatment initiation [12,27].

With the increasing use of SBRT, particularly in combination with modern systemic therapies, cancer care is progressively evolving toward a chronic disease model, with a growing number of patients achieving prolonged survival. Consequently, late treatment-related toxicities are becoming increasingly relevant in daily clinical practice. Although SBRT is generally associated with excellent local control and a favorable toxicity profile, severe late gastrointestinal complications, while rare [7,18,19], may substantially impair quality of life and significantly affect long-term outcomes.

In this context, HBOT may represent a promising supportive therapeutic option for selected patients with radiation-induced toxicity following SBRT. Nevertheless, robust clinical evidence remains limited, standardized treatment protocols are lacking, and access to HBOT is still restricted in many healthcare systems. Therefore, the management of complex SBRT-related toxicities requires a multidisciplinary approach involving radiation oncologists, gastroenterologists, hyperbaric medicine specialists, and supportive care teams, together with greater awareness and early recognition of potential late radiation-induced complications.

## 4. Conclusions

To the best of our knowledge, this represents the first reported case describing the successful use of HBOT as a potentially effective adjunctive treatment for severe radiation-induced duodenal ulceration following liver SBRT in a patient with cholangiocarcinoma. Although encouraging, this observation should be interpreted cautiously, and prospective clinical studies are needed to further evaluate the efficacy, safety, and optimal timing of HBOT in this setting.

## Data Availability

The original contributions presented in the study are included in the article; further inquiries can be directed to the corresponding author.
